# Information-Theoretic Cost–Benefit Analysis of Hybrid Decision Workflows in Finance

**DOI:** 10.3390/e27080780

**Published:** 2025-07-23

**Authors:** Philip Beaucamp, Harvey Maylor, Min Chen

**Affiliations:** 1 Department of Engineering Science, University of Oxford, Oxford OX1 3QG, UK; 2 Saïd Business School, University of Oxford, Oxford OX1 1HP, UK

**Keywords:** information theory, cost–benefit analysis, data-driven, decision-making, workflow, finance, incomplete information, knowledge, bias

## Abstract

Analyzing and leveraging data effectively has been an advantageous strategy in the management workflows of many contemporary organizations. In business and finance, data-informed decision workflows are nowadays essential for enabling development and growth. However, there is yet a theoretical or quantitative approach for analyzing the cost–benefit of the processes in such workflows, e.g., in determining the trade-offs between machine- and human-centric processes and quantifying biases. The aim of this work is to translate an information-theoretic concept and measure for cost–benefit analysis to a methodology that is relevant to the analysis of hybrid decision workflows in business and finance. We propose to combine an information-theoretic approach (i.e., information-theoretic cost–benefit analysis) and an engineering approach (e.g., workflow decomposition), which enables us to utilize information-theoretic measures to estimate the cost–benefit of individual processes quantitatively. We provide three case studies to demonstrate the feasibility of the proposed methodology, including (i) the use of a statistical and computational algorithm, (ii) incomplete information and humans’ soft knowledge, and (iii) cognitive biases in a committee meeting. While this is an early application of information-theoretic cost–benefit analysis to business and financial workflows, it is a significant step towards the development of a systematic, quantitative, and computer-assisted approach for optimizing data-informed decision workflows.

## 1. Introduction

Data plays a significant role in modern decision workflows. In business, for example, the competitive advantage of harnessing and analyzing data efficiently can be a critical factor in determining market leaders and laggards. Companies are increasingly employing data-informed strategies to enhance customer experiences, improve operational capabilities, and automate certain decision-making processes. Because such workflows commonly consist of various human- and machine-centric processes (e.g., data collection, statistical inference, machine learning, data mining, data visualization, report reading, discussion meetings, etc.), we intentionally use the term “*data-informed* decision workflows”, as the widely used adjective *data-driven* commonly implies automated machine-centric processes. While it is vital for organizations to adopt data-informed decision workflows [1], there are many challenges in designing, managing, and optimizing such data-informed decision workflows. For instance, (a) data collection may not be as comprehensive as desired due to various restrictions, (b) the quality of statistical inference may be affected by the sparsity and skewness of data, (c) the reliability of machine-learned models may be undermined by dated training data, (d) interpretation of visualization may be subjective, (e) human participants may introduce biases in discussions, and so forth. At the same time, all these component processes in data-informed decision workflows incur costs. Hence, there is a serious need for a scientific methodology that can be used to analyze, measure, and predict the performance of the component processes in a data-informed decision workflow. In this work, we propose a new methodology for using information to analyze and explain the cost–benefit of different processes in hybrid decision workflows in business and finance, where decisions rely on information from data and humans as well as technologies used. Our aim is to translate an information-theoretic concept and measure for cost–benefit analysis to a methodology that is relevant to business and finance. As the original concept is highly abstract, it is necessary to equip the methodology with an engineering approach for modeling and decomposing complex decision workflows into processes to which the concept can be applied in a quantitative manner.

We have tested this methodology in conjunction with a number of workflows in business, finance, and industry. In this paper, we present three case studies in a business and financial workflow, which represent three types of commonly encountered phenomena: (i) the use of a statistical and computational algorithm, (ii) the impact of incomplete information and humans’ soft knowledge, and (iii) the influence of cognitive biases in a committee meeting. These case studies demonstrate the feasibility of using the proposed methodology in analyzing and explaining the cost–benefit of different processes in data-informed decision workflows.

The remainder of the paper includes a brief overview of the relevant research in Section 2, a detailed description of the proposed methodology in Section 3, three case studies in Section 4, discussions on several related topics in Section 5, and our concluding remarks and suggestions for future work in Section 6.

## 2. Related Works

**Data-informed decision-making in companies.** In the foundational work “The Practice of Management”, Drucker [2] acknowledges the significance of data in influencing managerial decision-making to measure and enhance organizational performance. In a more recent study, Brynjolfsson et al. [3] put forth statistical evidence that data-informed decision-making significantly enhances productivity in modern firms. This finding is also confirmed by Provost and Fawcett [4], who strongly advocate for the positive effect of data-focused practices on organizational outcomes. Numerous scholars have concentrated on improving the utilization of data in enterprises over the last decades. Sun et al. [5] offer a data-flow framework, which focuses on data-flow specification and analysis, facilitating enhanced analytical rigor in business process management, particularly when encountering complex challenges. Further, Zhang and Perry [6] optimize time performance in business workflows using a data-centric approach to process modeling. Moreover, Kougka et al. [7] describe that data-centric workflow optimization undergoes a swift transformation and emphasize new capabilities of advanced data management for modern data-driven businesses, including big data analytics. Building on these studies, this work contributes to advancing data-informed decision-making in companies, illustrating a well-defined methodology to optimize business decision workflows.

**Human-centric elements in business data workflows.** Components in data intelligence processes can be machine- or human-centric [8]. Humans can enhance the scope of inference with “soft” information, which they can utilize in the form of pre-existing beliefs or knowledge. Tam et al. [9] conclude that human-centric designs of decision-tree models can outperform machine-centric approaches due to soft alphabets available to humans that are not present in data. Kijmongkolchai et al. [10] substantiate this finding with empirical evidence that humans use soft knowledge to reduce potential distortion. Our research incorporates the phenomena discussed in these previous studies into one information-theoretic framework. We utilize information theory to demonstrate that humans can mitigate potential distortion in a data-driven workflow through soft knowledge. At the same time, we demonstrate that human biases in decision-making can be effectively remedied through technology.

**Economic CBA methods.** Monetary cost–benefit analysis dates back more than a century. The net present value, as one of the most widely applied investment appraisal methods, was presented by Irving Fisher [11]. The Internal Rate of Return computes the discount rate that leads to a net present value of 0 of any given project and is widely used in private equity [12,13]. The Value of Information approximates the expected economic value of acquiring more information before a decision point [14]. Furthermore, the benefit–cost ratio, enables project evaluation by following the mathematical foundation of NPV to compare a project’s benefits and costs, albeit in a normalized form [15]. Whereas CAPEX and OPEX modeling, prevalent in project finance analysis, differentiate upfront investment from ongoing operational costs [16]. We will discuss the difference between economic CBA methods and the information-theoretic cost–benefit analysis in Section 5.

**Workflow optimization and decision process modeling.** The seminal work from Bellman (1957) introduces dynamic programming and Markovian decision processes (MDPs) to achieve optimal outcomes in multi-stage decision processes [17]. Kelley and Walker (1959) present the critical path method as a systematic technique to optimize a project schedule within a workflow [18]. Subsequently, Hammer and Champy (1993) put forth business process reengineering as a radical method for redesigning workflows [19]. Howard and Matheson (1984) utilize influence diagrams to represent the structure of complex decision problems under uncertainty [20]. In more contemporary work, Belousov and Peters (2019) extend MDP optimization by using information-theoretic regulation in the context of reinforcement learning [21]. Habermehl et al. (2022) connect model-based systems engineering with multidisciplinary design analysis and optimization to improve decision workflows in engineering [22]. This work builds upon the findings of these previous studies to develop an approach for analyzing data-informed decision workflows.

**Modern information-theoretical advances in explainable AI (XAI), bias control, and industrial digitization.** Multiple studies put forth evidence for the effective utilization of information-theoretical optimization in the context of explainable AI, bias control, and industrial digitization. Barbiero et al. (2022) extract explanations from neural networks, while optimizing an entropy-based criterion that determines the most pertinent concepts [23]. Sanneman (2024) describes an information-theoretic method, the Information Bottleneck approach, that optimizes for maximum informativeness and minimum complexity, thereby facilitating the design of user-specific XAI [24]. Mehdi and Tiwary (2024) present interpretation entropy as a general approach to assessing the human ability to interpret linear models [25]. Furthermore, Bang et al. (2021) present VIBI, the variational information bottleneck for interpretation, which utilizes the information bottleneck principle to identify concise yet extensive elaborations [26]. The contemporary review by Dutta and Haman (2023) emphasizes partial information decomposition as a strong information-theoretical tool to investigate explainability [27]. Kim and Cho (2022) present an information-theoretical method to alleviate bias in machine learning to achieve fairness [28]. Li et al. (2023) derive a non-vacuous upper bound based on information theory on the bias removal strength of any approach [29]. Similarly, substantial advances in entropy optimization are achieved in the field of industrial digitization. Omar and Plapper (2022) utilize path-transfer flow entropy to assess manufacturing flexibility quantitatively [30]. Herrera-Vidal et al. (2025) present an entropic metric of complexity for production systems developed based on simulation and programming methods [31]. Based on these previous studies, we present an operational method that utilizes decomposition and applies information-theoretic cost–benefit analysis for the optimization of data-informed decision-making workflows.

**Cost–benefit analysis of data intelligence workflows.** Chen and Jänicke [32] propose an information-theoretic framework of visualization, addressing the need to measure information content, loss and flow within pipelines and workflows. Chen and Golan [33] describe that processes within a data intelligence workflow can be subdivided into multiple steps, referred to as transformations. Further, building on literature on general information processing [34] and information-theoretical quantification in mathematics, economics, and statistics [35,36,37], Chen and Golan [33] present a cost–benefit measure, which can be utilized as a cost function to optimize data intelligence workflows. In other words, they provide the first measure that represents the trade-off between alphabet compression, potential distortion, and costs within a pipeline or data intelligence workflow. Subsequently, Chen and Ebert [38] outline the first systematic process for optimizing visual analytics workflows. Thereby, they analyze the weaknesses (symptoms) of the processes in a workflow and identified technical solutions (remedies) to improve the workflow. As the cost–benefit measure proposed by Chen and Golan [33] makes use of the Kullback–Leibler (KL) divergence [39], its potential distortion term is unbounded even when the total Shannon entropy [40] of the data space of a process is finite. Chen and Sbert [41] propose to use a bounded divergence measure for the potential distortion term. They studied several candidate measures conceptually, followed by several experiments in practical scenarios [42]. Furthermore, Chen [43] suggests applying the information-theoretic cost–benefit analysis to many data intelligence workflows in other fields, including machine learning, psychology, language development, and media communication.

## 3. Methodology

In this section, we first describe information-theoretic cost–benefit analysis, followed by a description of the workflow modeling and decomposition method. As mentioned in Section 1, using the latter (an engineering approach) to build a bridge from the former (a highly abstract concept) to hybrid decision workflows in business and finance is the aim and novel contribution of this work.

### 3.1. Information-Theoretic Cost–Benefit Analysis

A *data-informed workflow* consists of both human- and machine-centric processes that transform data into one or more decisions. In information theory, each process is represented by a function *F* referred to as a *transformation*, all possible inputs to *F* form an input space, and all possible outputs from *F* form an output space. The input and output spaces are represented by two alphabets, $Z_{\mathrm{in}}$ and $Z_{\mathrm{out}}$, respectively. All possible different inputs are the letters of $Z_{\mathrm{in}}$, which is associated with a probability mass function (PMF). Similarly, all possible different outputs are the letters of $Z_{\mathrm{out}}$. In general, a decision alphabet at the end of a data-informed workflow has much less entropy than the total entropy of all data alphabets that have contributed to the decision. Hence, there is usually a large entropy loss from data to decision.

Chen and Golan [33] first noticed the need to explain this ubiquitous phenomenon mathematically. They recognized that entropy loss, which is colloquially referred to as information loss, should not be regarded as a negative factor, since it is ubiquitous in data-informed workflows. They used two information-theoretic measures to represent the positive and negative aspects of entropy loss as *Alphabet Compression* and *Potential Distortion*. Together with a measure of cost, this gives the following conceptual formula:

(1) $$\frac{\mathrm{Benefit}}{\mathrm{Cost}}=\frac{\mathrm{Alphabet}\;\mathrm{Compression}\;\left(\mathrm{AC}\right)-\mathrm{Potential}\;\mathrm{Distortion}\;\left(\mathrm{PD}\right)}{\mathrm{Cos}t\;\left(\mathrm{Ct}\right)}$$

Given a transformation $F_{i}:Z_{i-1}\to Z_{i}$ (among a series of transformations), the term AC measures the entropy reduction achieved by a transformation $F_{i}$, i.e., $H\left(Z_{i-1}\right)-H\left(Z_{i}\right)$, where H is the measure of Shannon entropy [40]:

(2) $$H\left(Z\right)=H\left(P(Z)\right)=H\left(P\right)=-\sum_{i=1}^{n}p\left(z_{i}\right)\log_{2}p\left(z_{i}\right)$$

where the PMF of Z is $P\left(Z_{i}\right)=\left\{p\left(z_{1}\right),p\left(z_{2}\right),\ldots ,p\left(z_{n}\right)\right\}$. Since an input alphabet is more likely to have more entropy than an output alphabet, AC measures entropy loss positively. Note that with the $\log_{2}$ measure, H(Z) also has the unit “bit”.

The term PD measures the negative impact caused by the entropy loss due to the transformation $F_{i}$, such as errors of a recommendation algorithm or a human’s decision. Chen and Golan [33] proposed to use the KL divergence [39] for the PD term. Later, Chen and Sbert [42] proposed to use a bounded divergence measure instead, to make the AC and PD terms more comparable in scale. In this work, we adopt the bounded measure. Let *P* be the PMF of a reference alphabet $Z_{\mathrm{ref}}$ and *Q* be the PMF of a modeled or believed alphabet $Z_{\mathrm{bel}}$, the divergence measure proposed by Chen and Sbert is as follows:

(3) $$D=D_{\mathrm{cs}}\left(P\|Q\right)=\frac{1}{2}\sum_{i=1}^{n}\left(p_{i}+q_{i}\right)\log_{2}\left({\left|p_{i}-q_{i}\right|}^{2}+1\right)$$

with the $\log_{2}$ measure, $D_{\mathrm{cs}}\left(P∥Q\right)$ has the unit “bit”.

Chen and Sbert also considered that Jensen–Shannon divergence $D_{\mathrm{js}}$ could be a suitable bounded measure and discussed the relative merits with the aid of numerical experiments [42]. For consistency, we use a generic symbol D for the divergence measure in the next section, while using $D_{\mathrm{cs}}$ in all calculations. Readers can replace $D_{\mathrm{cs}}$ with $D_{\mathrm{js}}$ to observe slightly different behaviors of the $D_{\mathrm{js}}$ measure. As $D_{\mathrm{cs}}$ (as well as $D_{\mathrm{js}}$) is bounded by [0, 1], it is necessary to define PD in conjunction with a maximum entropy bound of the alphabet concerned. Since $Z_{\mathrm{ref}}$ and $Z_{\mathrm{bel}}$ share the same set of letters, we can use the maximum entropy of either.

Chen and Golan [33] considered that the ideal measure for the term cost would be energy, but recognized that, in practice, cost would typically be approximated by time, monetary cost, or another more attainable measure.

With the above definitions, the conceptual cost–benefit ratio in Equation (1) can be expressed quantitatively as follows:

(4) $$\frac{\mathrm{Benefit}}{\mathrm{Cost}}=\frac{H\left(P\right)-H\left(Q\right)-H_{\max}\left(Z_{\mathrm{ref}}\right)D\left(P∥Q\right)}{\mathrm{Cost}}$$

where *P* is the PMF of a reference alphabet $Z_{\mathrm{ref}}$ and *Q* is the PMF of a modeled or believed alphabet $Z_{\mathrm{bel}}$.

It is useful to emphasize that the cost–benefit measure is **reference-dependent**. When we measure the cost–benefit ratio, alphabet $Z_{\mathrm{ref}}$ can be a confirmed ground truth, which leads to the actual distortion incurred with $Z_{\mathrm{bel}}$. However, in practice, we may use an estimated or presumed ground truth, and in such a case, when D(P∥Q)>0, it does not necessarily imply $Z_{\mathrm{bel}}$ is actually incorrect. **$Z_{\mathrm{bel}}$ is seen to cause a distortion only from the reference point of $Z_{\mathrm{ref}}$**. Hence, the terms “reference alphabet” and “potential distortion” are here to imply that $Z_{\mathrm{ref}}$ is not necessarily a ground truth and D(P∥Q) measures only the potential distortion. In Section 4, we will use $Z_{\mathrm{ref}}$ in different contexts. Because of the reference point, the cost–benefit measure is also reference-dependent.

As this work focuses on the translation of the abstract information-theoretic concept and the cost–benefit measure in Equation (4), we assume that the letters of each alphabet have already been defined and all PMFs have already been obtained. There is no constraint as to the distribution that a PMF may take. In Section 5, we will discuss the relevance of the existing and future research on PMF estimation, uncertainty analysis, and sensitivity analysis to further advancement in transforming this work to real-world applications.

### 3.2. Workflow Decomposition

Although the information-theoretic cost–benefit analysis has been used to explain why data visualization is a cost-beneficial tool in general [33] and to measure simple visualization processes [41,42], it has not yet been used to analyze any slightly complex workflow quantitatively. There were suggestions that such applications may be possible [43], but the attempts reported in the literature are largely qualitative methods based on Equation (1) (e.g., [38]). The main reason behind the lack of reported quantitative attempts is that for any slightly complex data-informed decision process (i.e., a transformation in terms of information theory), the input data space is usually large and very complex, which translates to a situation where the corresponding input alphabet has numerous letters and the PMF for such an alphabet is usually very difficult to estimate.

To address this challenge, we adopt the common engineering approach for decomposing a complex data-informed decision workflow into a series of processes. If any processes are still too complex to be analyzed using the information-theoretic cost–benefit analysis, we can further decompose these processes iteratively, until the input and output alphabets for each process are reasonably easy to define and the corresponding PMFs are relatively easy to estimate. Of course, one could end up with dozens or even hundreds of processes, which might sound unmanageable. However, in many engineering disciplines (e.g., the design and management of power or chemical plants), engineers have commonly dealt with workflows involving hundreds or thousands of processes, even before computers were used to aid such tasks. Hence, it will be feasible to analyze many processes in a complex data-informed decision workflow if we can develop a generalizable method to analyze some individual processes. This is precisely the focus of this work. We believe that as this generalizable method matures, computer-aided tools will emerge to help human analysts manage hundreds or thousands of processes, as is common in many engineering disciplines today.

**Figure 1:**
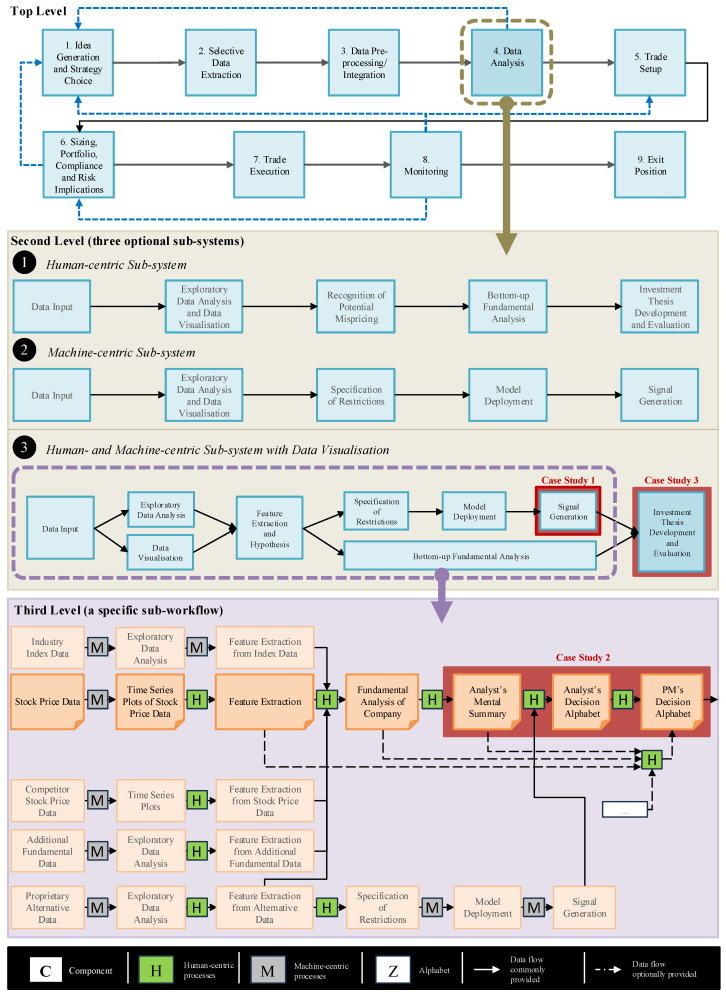
Decomposition of a data-informed decision workflow for discretionary long/short equity trading.

For example, consider a complex data-informed decision workflow at the top of Figure 1. This is a representative workflow for making discretionary long/short equity decisions in many financial organizations. At the top level, there are nine processes, each of which is still too complex to be analyzed using the information-theoretic cost–benefit analysis. We can decompose any of these processes further. For instance, if we are interested in process “4. Data Analysis”, we can consider three design options in the middle of Figure 1, labeled with black-circled numbers 1, 2, and 3, respectively. (1) A human-centric sub-system represents an old practice without using many data science tools. (2) A machine-centric sub-system represents a series of processes for making decisions automatically, which many consider to be optimal in the future. (3) A sub-system, where human- and machine-centric processes are integrated together and make decisions, represents the most common practice today.

Let us focus on the sub-system (3), where we can identify process blocks that are small enough for information-theoretic cost–benefit analysis (as opposed to others that will need further decomposition). The decomposition of one such block is shown at the bottom part of Figure 1. In the next section, we show how the processes in three case studies can be analyzed using the method described in Section 3.1. These three case studies are marked in Figure 1 with red boxes.

## 4. Case Studies

In the following section, we illustrate the feasibility of using cost–benefit analysis for machine-centric and human-centric decision processes through three case studies, each demonstrating selective facets of the method. The abstraction describes a data-informed decision workflow with machine- and human-centric components, examining an exemplary long/short equity strategy. We compare various process types, such as parallel and sequential processes, as well as the level of requested information. Furthermore, we focus on the human-centric components of the investment committee and suggest improvements to ensure the effective execution of the meeting process. Similarly, we estimate the amount of alphabet compression, potential distortion, and cost of selected components. We consider some typical phenomena in such a workflow, including consistent data analysis or erroneous recommendations by algorithms, appropriate or inappropriate use of visualization, expert knowledge or personal biases of committee members, missing data, and so forth. Finally, we analyze the internal policy and cost considerations of involving an investment committee or executing a trade without further external scrutiny. In the first case study, we demonstrate the analysis of machine-centric processes, illustrated by examining an analytical moving average (MA) crossover algorithm. In the second and third case studies, we examine the influence of incomplete information, soft knowledge, human bias and cost on the workflow structure and decision outcomes.

### 4.1. Case Study 1: An Analytical Algorithm Scenario

In business and financial decision workflows, there are many computational processes. Intuitively, we all know that these computational processes can significantly reduce the cost. Information-theoretically, they also typically reduce a significant amount of entropy from input to output, thereby facilitating alphabet compression. For example, in a financial decision-making workflow of a long/short equity hedge fund, many analytical algorithms facilitate trading decisions by utilizing quantitative signals derived from a multitude of data sources, such as industry index data, stock price data, and proprietary alternative data. One algorithm is frequently used for monitoring *moving average crossovers*—a well-established technical momentum indicator. In Figure 1, the block marked as “Case Study 1” commonly includes the use of the *moving average crossovers* method.

Consider an analyst who utilizes a crossover algorithm to compare 50-day and 200-day moving averages for *M* equities, over a period *T*. When the 50-day average **rises above** (or **falls below**) the 200-day average for any single stock, the algorithm triggers a **bullish** (or **bearish**) signal and recommends a **long** (or **short**) position due to **positive** (or **negative**) momentum. While a systematic trading algorithm could execute a **long** (or **short**) position based on this signal alone, its utilization varies among hedge funds. Nevertheless, one can reasonably assume that these crossover indicators allow the analyst to identify potentially interesting trading opportunities for further analysis and enable more efficient monitoring of stocks. Let the period *T* be sampled as $t_{1},t_{2},\ldots ,t_{n}$. Let $s_{k}$ be one of the *M* stocks covered by the analyst. The moving average crossover signal is a ternary time series for the stock $\sigma_{1}\left(s_{k}\right),\sigma_{2}\left(s_{k}\right),\ldots ,\sigma_{n}\left(s_{k}\right)$, such that

(5) $$\sigma_{t}\left(s_{k}\right)=\left\{\begin{array}{cc} +1, & \mathrm{if}\;{\mathrm{MA}}_{50}\left(t-1,s_{k}\right)\leq {\mathrm{MA}}_{200}\left(t-1,s_{k}\right)\;\mathrm{and}\;{\mathrm{MA}}_{50}\left(t,s_{k}\right)>{\mathrm{MA}}_{200}\left(t,s_{k}\right)\\ -1, & \mathrm{if}\;{\mathrm{MA}}_{50}\left(t-1,s_{k}\right)\geq {\mathrm{MA}}_{200}\left(t-1,s_{k}\right)\;\mathrm{and}\;{\mathrm{MA}}_{50}\left(t,s_{k}\right)<{\mathrm{MA}}_{200}\left(t,s_{k}\right)\\ 0, & \mathrm{otherwise} \end{array}\right.$$

where ${\mathrm{MA}}_{50}\left(t,s_{k}\right)$ and ${\mathrm{MA}}_{200}\left(t,s_{k}\right)$ are, respectively, the 50-day and 200-day moving averages of stock $s_{k}$ at time *t* [44].

In the context of an individual temporal point *t*, the original stock value is represented by a 32-bit integer [0, 4,294,967,295]. (To date, the highest share value recorded is less than 100,000,000 cents.) Let $Z_{\mathrm{sv}}$ be the alphabet for representing share values in a 32-bit integer, and the maximum entropy of $Z_{\mathrm{sv}}$ is thus 32 bits. Even if we consider only the value range [0, 100,000,000], the maximum entropy would still be about 26.575 bits. As very high share values are rare, the typical entropy of $Z_{\mathrm{sv}}$ is much lower than its maximum entropy. Meanwhile, to encode $\sigma_{t}\left(s_{k}\right)$, one needs an alphabet, $Z_{\sigma }$, with only three letters (+1,−1,0), and its maximum entropy is about 1.585 bits. Likely, the probability mass function (PMF) is in favor of 0 at most time steps, and the entropy of $Z_{\sigma }$ is much lower than 1.585 bits.

To simplify the discussions below, we assume that the time steps in the period *T* are the intervals between two consecutive trading days. To derive a single $\sigma_{t}\left(s_{k}\right)$ indicator, the algorithm needs 200 days of stock values of $s_{k}$. To derive *n*$\sigma_{t}\left(s_{k}\right)$ indicators for $t_{1},t_{2},\ldots ,t_{n}$, the algorithm needs n+199 days of stock values of $s_{k}$. If we estimate the **alphabet compression** of this transformation using maximum entropy, we have the following:

$$\mathrm{MA}\;\mathrm{Crossover}\;\mathrm{Transformation}:\left(n+199\right)\;H_{\max}\left(Z_{\mathrm{sv}}\right)-n\;H_{\max}\left(Z_{\sigma }\right)$$

For n=100 days, this amounts to 299×32−100×1.585=9409.5 bits per stock. We can consider this as an upper bound. To estimate a lower bound, we may assume that the entropy of an individual share is about 6 bits (up to 64 different values uniformly distributed). With such an assumption, for n=100 days, it amounts to 299×6−100×1.585=1635.5 bits per stock. We, thus, have the lower and upper bounds for alphabet compression per stock as [1635.5, 9409.5] bits. When we consider the analyst has to cover *M* equities, the amount of alphabet compression is even more impressive.

With such a considerable amount of alphabet compression, the transformation will no doubt incur a large amount of **potential distortion** if one is required to use a single indicator $\sigma_{t}\left(s_{k}\right)$ to reconstruct a 200-point time series of the share values of $s_{k}$, or the time series representing the long- and short-term moving averages. The task is unattainable due to the unusable and erroneous outcome, as well as the unaffordable cognitive effort. However, this is hardly ever necessary. The time series plot for the original data and moving averages is usually readily available, and the analyst can simply glance at the plot to regain information about the original time series and the moving average lines. In this way, the reconstruction accuracy is reasonably high, and the **cost** is very low. Let us consider the following:

- **AC**—The amount of Alphabet Compression achieved by the MA Crossover Algorithm;
- **PD-noVis**—The amount of potential distortion if one does not visualize any of the time series (i.e., the original and moving averages);
- **PD-Vis**—The amount of potential distortion if one does visualize the time series (i.e., the original and moving averages);
- **Cost-noVis**—The amount of cognitive effort for reconstructing any of the time series from the MA Crossover indicators (or the cost of decision errors due to not being able to view any of the time series);
- **Cost-Vis**—The amount of cognitive effort for viewing the time series (or the cost of decision errors after viewing the time series);

We can easily conclude:

$$\frac{\mathrm{benefit}}{\mathrm{cost}}:\;\frac{\mathrm{AC}-\mathrm{PD-noVis}}{\mathrm{Cost-noVis}}\ll \frac{\mathrm{AC}-\mathrm{PD-Vis}}{\mathrm{Cost-Vis}}$$

The information-theoretic analysis of this scenario applies to many uses of statistical and computational methods in business and financial workflows and can explain why financial analysts and managers frequently glance at time series plots and other data visualization plots.

### 4.2. Case Study 2: Two Analyst–Portfolio Manager Scenarios

In business and financial decision workflows, it is common to have a process where an analyst proposes a decision and a manager confirms, rejects, or modifies the decision. Figure 1 shows such an example process (marked as “Case Study 2”), where an analyst obtains conventional financial data, alternative data, internal data, third-party data, *inter alia*, to assess equity investment opportunities. A portfolio manager receives a recommendation from the analyst and makes the final decision. In such processes, critical information may often not be captured by the available data. When an analyst presents a trade idea and the corresponding investment thesis, the recommendation may or may not be appropriate, depending on how the analyst addresses the missing information. Meanwhile, a diligent portfolio manager may pay attention to the risk due to missing information, discussing how the analyst’s knowledge was used to make assumptions or derive inferences from other information. In contrast, an inattentive portfolio manager may simply accept the analyst’s recommendation at face value. Let us consider two scenarios:

- **CS2x**—Inappropriate Recommendation, Diligent Manager
- **CS2y**—Inappropriate Recommendation, Inattentive Manager

**Figure 2:**
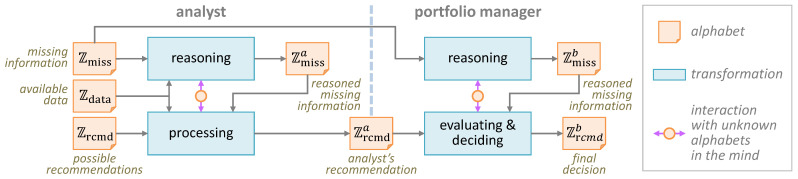
The detailed alphabets and transformations in the “Case Study 2” part of the workflow in Figure 1.

Consider the detailed diagram illustrating this process in Figure 2, where alphabet $Z_{\mathrm{data}}$ consists of all possible variants of the data that the analyst has processed and alphabet $Z_{\mathrm{miss}}$ consists of all possible variants of the missing information. $Z_{\mathrm{miss}}^{a}$ is the alphabet of missing information after the analyst has reasoned with assumptions and inference, while $Z_{\mathrm{miss}}^{b}$ is the alphabet of missing information after the manager has considered the analyst’s reasoning together with the manager’s own assumptions and inference. Along the recommendation flow, the alphabet $Z_{\mathrm{rcmd}}$ consists of all possible recommendations that the analyst could make before processing the data. $Z_{\mathrm{rcmd}}^{a}$ denotes the alphabet after a recommendation has been formulated, and $Z_{\mathrm{rcmd}}^{b}$ is the alphabet representing the portfolio manager’s final decision on the recommendation.

Information-theoretically, we can first recognize the amount of alphabet compression that has been achieved by the analyst who transforms the three alphabets on the left to the alphabet of the analyst’s recommendation, since $Z_{\mathrm{data}}$, $Z_{\mathrm{miss}}$, and $Z_{\mathrm{rcmd}}$ should have much more entropy than $Z_{\mathrm{rcmd}}^{a}$. Typically, the recommendation alphabet $Z_{\mathrm{rcmd}}$ consists of two sub-alphabets. One has 75 letters for encoding different summary views on stock direction, catalyst existence, and conviction, i.e.,

$$Z_{\mathrm{rcmd}1}=\left\{\begin{array}{c} \mathrm{strong}\mathrm{rise}\\ \mathrm{rise}\\ \mathrm{neutral}\\ \mathrm{decline}\\ \mathrm{strong}\mathrm{decline} \end{array}\right\}\times \left\{\begin{array}{c} \mathrm{only}\mathrm{one}\mathrm{short-term}\mathrm{catalyst}\\ \mathrm{only}\mathrm{one}\mathrm{medium-term}\mathrm{catalyst}\\ \mathrm{only}\mathrm{one-long}\mathrm{term}\mathrm{catalyst}\\ \mathrm{multiple}\mathrm{catalysts}\\ \mathrm{no}\mathrm{catalysts} \end{array}\right\}\times \left\{\begin{array}{c} \mathrm{high}\mathrm{conviction}\\ \mathrm{medium}\mathrm{conviction}\\ \mathrm{low}\mathrm{conviction} \end{array}\right\}$$

The other consists of four letters representing possible recommended decisions on a summary view:

$$Z_{\mathrm{rcmd}2}=\left\{\mathrm{accept},\;\mathrm{reject},\;\mathrm{request}\;\mathrm{more}\;\mathrm{information},\;\mathrm{wait}\right\}$$

Its maximum entropy is $H_{\max}\left(Z_{\mathrm{rcmd}}\right)\approx 8.2$ bits. As the PMF of $Z_{\mathrm{rcmd}}$ (before processing) is expected to be less certain than the PMF of $Z_{\mathrm{rcmd}}^{a}$ (after processing), we have $H\left(Z_{\mathrm{rcmd}}\right)>H\left(Z_{\mathrm{rcmd}}^{a}\right)$. Meanwhile, alphabets $Z_{\mathrm{data}}$ and $Z_{\mathrm{miss}}$ are defined upon many complex variables, including time series variables and multivariate factual data, their maximum entropy is likely to be of hundreds of bits or more. The alphabet compression achieved by the analyst can reduce the cost of subsequent processes, including but not limited to the manager’s effort for processing data.

There are many factors that may affect the quality of the analyst’s recommendation $Z_{\mathrm{rcmd}}^{a}$, including the analyst’s knowledge and experience, the critical nature of the missing information, and the access to data analysis and visualization tools. An inattentive portfolio manager may simply adopt the analyst’s recommendation. For example, an analyst may present a summary view (*strong rise*, *multiple catalysts*, *high conviction*) and then express “*I’m 90% sure that the summary view is correct, though I am also OK to reject.*” An inattentive portfolio manager makes a quick decision to accept the summary view. In terms of the alphabets in Figure 2, we have the PMFs:

$$\begin{array}{cc} P(Z_{\mathrm{rcmd}1}^{a}) & =\left\{\begin{array}{cc} 1 & \mathrm{letter}\;(\mathrm{strong}\;\mathrm{rise},\;\mathrm{multiple}\;\mathrm{catalysts},\;\mathrm{high}\;\mathrm{conviction})\\ 0 & \mathrm{other}\;74\;\mathrm{letters} \end{array}\right.\\ P(Z_{\mathrm{rcmd}2}^{a}) & =\left\{\begin{array}{cc} 0.9 & \mathrm{letter}\;\mathrm{accept}\\ 0.1 & \mathrm{letter}\;\mathrm{reject}\\ 0 & \mathrm{other}\;2\;\mathrm{letters}\; \end{array}\right.\;P\left(Z_{\mathrm{rcmd}2}^{b}\right)=\left\{\begin{array}{cc} 1 & \mathrm{letter}\;\mathrm{accept}\\ 0 & \mathrm{letter}\;\mathrm{reject}\\ 0 & \mathrm{other}\;2\;\mathrm{letters}\; \end{array}\right. \end{array}$$

A diligent portfolio manager evaluates the analyst’s recommendation more carefully, reasons about some or all missing information independently, and makes the final decision that may differ from the analyst’s recommendation, e.g.,

$$\begin{array}{cc} P(Z_{\mathrm{rcmd}1}^{b}) & =\left\{\begin{array}{cc} 0.4 & \mathrm{letter}\;(\mathrm{strong}\;\mathrm{rise},\;\mathrm{multiple}\;\mathrm{catalysts},\;\mathrm{medium}\;\mathrm{conviction})\\ 0.6 & \mathrm{letter}\;(\mathrm{rise},\;\mathrm{multiple}\;\mathrm{catalysts},\;\mathrm{medium}\;\mathrm{conviction})\\ 0 & \mathrm{other}\;73\;\mathrm{letters} \end{array}\right.\\ P(Z_{\mathrm{rcmd}2}^{b}) & =\left\{\begin{array}{cc} 1 & \mathrm{letter}\;\mathrm{accept}\\ 0 & \mathrm{other}\;3\;\mathrm{letters}\; \end{array}\right. \end{array}$$

Assume that a post hoc analysis shows an ideal recommendation $Z_{\mathrm{rcmd}}^{*}$ such that

$$\begin{array}{cc} P(Z_{\mathrm{rcmd}1}^{*}) & =\left\{\begin{array}{cc} 1 & \mathrm{letter}\;(\mathrm{rise},\;\mathrm{multiple}\;\mathrm{catalysts},\;\mathrm{medium}\;\mathrm{conviction})\\ 0 & \mathrm{other}\;74\;\mathrm{letters} \end{array}\right.\\ P(Z_{\mathrm{rcmd}2}^{*}) & =\left\{\begin{array}{cc} 1 & \mathrm{letter}\;\mathrm{accept}\\ 0 & \mathrm{other}\;3\;\mathrm{letters}\; \end{array}\right. \end{array}$$

We can consider that $Z_{\mathrm{rcmd}}^{*}$ represents the ground truth, and it could be discovered by an ideal process (e.g., without missing information). We can also consider that $Z_{\mathrm{rcmd}}$ is a masked version of $Z_{\mathrm{rcmd}}^{*}$. The goal of the process in Figure 2 is thus to reconstruct $Z_{\mathrm{rcmd}}^{*}$, i.e.,

$$Z_{\mathrm{rcmd}}^{*}\;\underset{\to }{\;\mathrm{disguising}\;}\;Z_{\mathrm{rcmd}}\;\underset{\to }{\;\mathrm{processing}\;}\;Z_{\mathrm{rcmd}}^{a}\;\underset{\to }{\;\mathrm{evaluating}\;}\;Z_{\mathrm{rcmd}}^{b}$$

Note that this sequence of transformations is very similar to those found in typical empirical studies in psychology. For such a sequence, we can evaluate the **potential distortion** of the analyst’s recommendation and the diligent portfolio manager’s final decision. For example, using the above $Z_{\mathrm{rcmd}}^{*}$, $Z_{\mathrm{rcmd}}^{a}$, and $Z_{\mathrm{rcmd}}^{b}$, we have the following:

$$\begin{array}{cc} \mathrm{analyst}: & \;H_{\max}\left(Z_{\mathrm{rcmd}}^{*}\right)D\left(Z_{\mathrm{rcmd}}^{*}∥Z_{\mathrm{rcmd}}^{a}\right)\approx 8.229\times 0.886\approx 7.290\;\mathrm{bits}\\ \mathrm{inattentive}\mathrm{portfolio}\mathrm{manager}: & \;H_{\max}\left(Z_{\mathrm{rcmd}}^{*}\right)D\left(Z_{\mathrm{rcmd}}^{*}∥Z_{\mathrm{rcmd}}^{b}\right)\approx 8.229\times 1.000\approx 8.229\;\mathrm{bits}\\ \mathrm{diligent}\mathrm{portfolio}\mathrm{manager}: & \;H_{\max}\left(Z_{\mathrm{rcmd}}^{*}\right)D\left(Z_{\mathrm{rcmd}}^{*}∥Z_{\mathrm{rcmd}}^{b}\right)\approx 8.229\times 0.214\approx 1.762\;\mathrm{bits} \end{array}$$

Let the PMF of the disguised alphabet $Z_{\mathrm{rcmd}}$ be uniformly distributed. We can also calculate the **alphabet compression** achieved by the analyst, and the inattentive and the diligent portfolio managers:

$$\begin{array}{cc} \mathrm{analyst}: & \;H\left(Z_{\mathrm{rcmd}}\right)-H\left(Z_{\mathrm{rcmd}}^{a}\right)\approx 8.229-0.469\approx 7.760\;\mathrm{bits}\\ \mathrm{inattentive}\mathrm{portfolio}\mathrm{manager}: & \;H\left(Z_{\mathrm{rcmd}}\right)-H\left(Z_{\mathrm{rcmd}}^{b}\right)\approx 8.229-0.000\approx 8.229\;\mathrm{bits}\\ \mathrm{diligent}\mathrm{portfolio}\mathrm{manager}: & \;H\left(Z_{\mathrm{rcmd}}\right)-H\left(Z_{\mathrm{rcmd}}^{b}\right)\approx 8.229-0.971\approx 7.258\;\mathrm{bits} \end{array}$$

We can calculate the informative **benefit** as follows:

$$\begin{array}{cc} \mathrm{analyst}: & \;7.760-7.290\approx 0.470\;\mathrm{bits}\\ \mathrm{inattentive}\mathrm{portfolio}\mathrm{manager}: & \;8.229-8.229=0\;\mathrm{bits}\\ \mathrm{diligent}\mathrm{portfolio}\mathrm{manager}: & \;7.258-1.762\approx 5.496\;\mathrm{bits} \end{array}$$

We can also estimate the costs of an analyst and a portfolio manager in terms of the amount of time they would be expected to perform the tasks concerned. For example, one may expect an analyst to spend 5 h and a portfolio manager to spend 1 h on the left and right parts of the tasks in Figure 2. We can estimate the cost benefit as follows:

*Analyst**Inattentive PM**Diligent PM*$\frac{\mathrm{Benefit}}{\mathrm{Cost}}$$\frac{0.470}{5}=0.094$$\frac{\mathrm{bits}}{\mathrm{hour}}$$\frac{0}{1}=0$$\frac{\mathrm{bits}}{\mathrm{hour}}$$\frac{5.496}{1}=5.496$$\frac{\mathrm{bits}}{\mathrm{hour}}$

### 4.3. Case Study 3: An Investment Committee Scenario

Following the portfolio manager’s decision to accept an investment thesis, it is frequently presented to an investment committee for approval. In Figure 1, this would be conducted as part of the Investment Thesis Development and Evaluation component. While members of the investment committee are typically experienced portfolio managers, they may have different specialities. Some may focus on equities within industries less relevant to the presented investment thesis. In Figure 1, this case study is labeled as “Case Study 3”. At this point in the pipeline, most funds have a policy regarding the mandatory involvement of an investment committee, which provides an additional governance layer and offers domain-specific expertise, soft knowledge, and risk evaluation.

Let us consider a scenario in which an investment committee is tasked with evaluating a recommendation by a portfolio manager in Case Study 2. The investment committee needs to make a decision, the alphabet of which is $Z_{\mathrm{cmdc}}$. Similar to $Z_{\mathrm{rcmd}}$, $Z_{\mathrm{cmdc}}$ consists of two sub-alphabets, $Z_{\mathrm{cmdc}1}$ and $Z_{\mathrm{cmdc}2}$. $Z_{\mathrm{cmdc}1}$ has the same letters as $Z_{\mathrm{rcmd}1}$, while $Z_{\mathrm{cmdc}2}$ has three of the four letters in $Z_{\mathrm{rcmd}2}$, i.e.,

$$Z_{\mathrm{cmdc}2}=\left\{\mathrm{accept},\;\mathrm{reject},\;\mathrm{request}\;\mathrm{more}\;\mathrm{information}\right\}$$

The maximum entropy is $H_{\max}\left(Z_{\mathrm{cmdc}}\right)\approx 7.8$ bits. Consider a case where a portfolio manager, **PM**, presents a proposal, $Z_{\mathrm{cmdc}}^{\mathrm{PM}}$, to the committee before the committee meeting:

$$\begin{array}{cc} P(Z_{\mathrm{cmdc}1}^{\mathrm{PM}}) & =\left\{\begin{array}{cc} 0.8 & \mathrm{letter}\;(\mathrm{strong}\;\mathrm{decline},\;\mathrm{one}\;\mathrm{short-term}\;\mathrm{catalyst},\;\mathrm{high}\;\mathrm{conviction})\\ 0.2 & \mathrm{letter}\;(\mathrm{strong}\;\mathrm{decline},\;\mathrm{only}\;\mathrm{one}\;\mathrm{medium-term}\;\mathrm{catalyst},\;\mathrm{high}\;\mathrm{conviction})\\ 0 & \mathrm{other}\;73\;\mathrm{letters} \end{array}\right.\\ P(Z_{\mathrm{cmdc}2}^{\mathrm{PM}}) & =\left\{\begin{array}{cc} 1 & \mathrm{letter}\;\mathrm{accept}\\ 0 & \mathrm{other}\;2\;\mathrm{letters}\; \end{array}\right. \end{array}$$

At the beginning of the committee meeting, we have

$$Z_{\mathrm{cmdc}}^{0}=\left[\begin{array}{c} Z_{\mathrm{cmdc}1}^{0}\\ Z_{\mathrm{cmdc}2}^{0} \end{array}\right]=\left[\begin{array}{c} Z_{\mathrm{cmdc}1}^{\mathrm{PM}}\\ \left\{\mathrm{accept}(p=0.6),\mathrm{reject}(p=0.3),\mathrm{request}\mathrm{more}\mathrm{information}(p=0.1)\right\} \end{array}\right]$$

where the PMF (0.6, 0.3, 0.1) reflects the historical statistics of the committee’s decisions. Note that $P\left(Z_{\mathrm{cmdc}1}^{\mathrm{PM}}\right)=P\left(Z_{\mathrm{cmdc}1}^{0}\right)$, but $P\left(Z_{\mathrm{cmdc}2}^{\mathrm{PM}}\right)\neq P\left(Z_{\mathrm{cmdc}2}^{0}\right)$.

Consider a committee that has four members:

- **PM**—The portfolio manager who proposed the recommendation.
- **MS**—A member of the committee with useful soft knowledge.
- **MP**—A member of the committee with positive bias.
- **MN**—A member of the committee with negative bias.

We also assume that the ground truth of $Z_{\mathrm{cmdc}1}^{*}$ and the ideal decision $P(Z_{\mathrm{cmdc}2}^{*})$ are as follows:

$$\begin{array}{cc} P(Z_{\mathrm{cmdc}1}^{*}) & =\left\{\begin{array}{cc} 1 & \mathrm{letter}\;(\mathrm{rise},\;\mathrm{multiple}\;\mathrm{catalysts},\;\mathrm{medium}\;\mathrm{conviction})\\ 0 & \mathrm{other}\;74\;\mathrm{letters} \end{array}\right.\\ P(Z_{\mathrm{cmdc}2}^{*}) & =\left\{\begin{array}{cc} 1 & \mathrm{letter}\;\mathrm{reject}\\ 0 & \mathrm{other}\;2\;\mathrm{letters}\; \end{array}\right. \end{array}$$

The member of the committee, **MS**, with a strong informational network, pattern recognition capability, and routine (i.e., soft knowledge), expresses a view that is represented by the alphabet $Z_{\mathrm{cmdc}}^{\mathrm{MS}}$:

$$\begin{array}{cc} P(Z_{\mathrm{cmdc}1}^{\mathrm{MS}}) & =\left\{\begin{array}{cc} 0.9 & \mathrm{letter}\;(\mathrm{rise},\;\mathrm{multiple}\;\mathrm{catalysts},\;\mathrm{medium}\;\mathrm{conviction})\\ 0.1 & \mathrm{letter}\;(\mathrm{rise},\;\mathrm{multiple}\;\mathrm{catalysts},\;\mathrm{low}\;\mathrm{conviction})\\ 0 & \mathrm{other}\;73\;\mathrm{letters} \end{array}\right.\\ P(Z_{\mathrm{cmdc}2}^{\mathrm{MS}}) & =\left\{\begin{array}{cc} 1 & \mathrm{letter}\;\mathrm{reject}\\ 0 & \mathrm{other}\;2\;\mathrm{letters}\; \end{array}\right. \end{array}$$

This is fairly close to $Z_{\mathrm{cmdc}}^{*}$.

One member, **MP**, who has advised on the investment thesis before the committee and who holds an overall negative view of the industry, exhibits a strongly positive bias in favor of the trade with $Z_{\mathrm{MP}}^{\mathrm{cmdc}}$:

$$\begin{array}{cc} P(Z_{\mathrm{cmdc}1}^{\mathrm{MP}}) & =\left\{\begin{array}{cc} 1 & \mathrm{letter}\;(\mathrm{strong}\;\mathrm{decline},\;\mathrm{one}\;\mathrm{short-term}\;\mathrm{catalyst},\;\mathrm{high}\;\mathrm{conviction})\\ 0 & \mathrm{other}\;74\;\mathrm{letters} \end{array}\right.\\ P(Z_{\mathrm{cmdc}2}^{\mathrm{MP}}) & =\left\{\begin{array}{cc} 1 & \mathrm{letter}\;\mathrm{accept}\\ 0 & \mathrm{other}\;2\;\mathrm{letters}\; \end{array}\right. \end{array}$$

Another member, **MN**, offers a view at a later stage, exhibiting a strong negative bias against the trade. Such cognitive bias may stem from the explicit competition for capital allocation or the need to enhance the relative merit of their trade, resulting in $Z_{\mathrm{cmdc}}^{\mathrm{MN}}$:

$$\begin{array}{cc} P(Z_{\mathrm{cmdc}1}^{\mathrm{MN}}) & =\left\{\begin{array}{cc} 0.5 & \mathrm{letter}\;(\mathrm{rise},\;\mathrm{multiple}\;\mathrm{catalysts},\;\mathrm{high}\;\mathrm{conviction})\\ 0.5 & \mathrm{letter}\;(\mathrm{rise},\;\mathrm{multiple}\;\mathrm{catalysts},\;\mathrm{medium}\;\mathrm{conviction})\\ 0 & \mathrm{other}\;73\;\mathrm{letters} \end{array}\right.\\ P(Z_{\mathrm{cmdc}2}^{\mathrm{MN}}) & =\left\{\begin{array}{cc} 1 & \mathrm{letter}\;\mathrm{reject}\\ 0 & \mathrm{other}\;2\;\mathrm{letters}\; \end{array}\right. \end{array}$$

We can compute the **alphabet compression** achieved by each committee member as if they do not know $Z_{\mathrm{cmdc}}^{0}$ (including **PM**):

$$\begin{array}{cc} \mathrm{PM}(\mathrm{the}\mathrm{proposer}): & \;H_{\max}\left(Z_{\mathrm{cmdc}}\right)-H\left(Z_{\mathrm{cmdc}}^{\mathrm{PM}}\right)\approx 7.814-0.722\approx 7.092\;\mathrm{bits}\\ \mathrm{MS}(\mathrm{with}\mathrm{soft}\mathrm{knowledge}): & \;H_{\max}\left(Z_{\mathrm{cmdc}}\right)-H\left(Z_{\mathrm{cmdc}}^{\mathrm{MS}}\right)\approx 7.814-0.469\approx 7.345\;\mathrm{bits}\\ \mathrm{MP}(\mathrm{with}\mathrm{positive}\mathrm{bias}): & \;H_{\max}\left(Z_{\mathrm{cmdc}}\right)-H\left(Z_{\mathrm{cmdc}}^{\mathrm{MP}}\right)\approx 7.814-0.000\approx 7.814\;\mathrm{bits}\\ \mathrm{MN}(\mathrm{with}\mathrm{negative}\mathrm{bias}): & \;H_{\max}\left(Z_{\mathrm{cmdc}}\right)-H\left(Z_{\mathrm{cmdc}}^{\mathrm{MN}}\right)\approx 7.814-1.000\approx 6.814\;\mathrm{bits} \end{array}$$

However, if the views of **MS**, **MP**, and **MN** are formulated after seeing the proposal of **PM**, we compute the **alphabet compression** as follows:

$$\begin{array}{cc} \mathrm{PM}(\mathrm{the}\mathrm{proposer}): & \;H\left(Z_{\mathrm{cmdc}}^{\mathrm{PM}}\right)-H\left(Z_{\mathrm{cmdc}}^{\mathrm{PM}}\right)\approx 0.722-0.722=0\;\mathrm{bits}\\ \mathrm{MS}(\mathrm{with}\mathrm{soft}\mathrm{knowledge}): & \;H\left(Z_{\mathrm{cmdc}}^{\mathrm{PM}}\right)-H\left(Z_{\mathrm{cmdc}}^{\mathrm{MS}}\right)\approx 0.722-0.469\approx 0.253\;\mathrm{bits}\\ \mathrm{MP}(\mathrm{with}\mathrm{positive}\mathrm{bias}): & \;H\left(Z_{\mathrm{cmdc}}^{\mathrm{PM}}\right)-H\left(Z_{\mathrm{cmdc}}^{\mathrm{MP}}\right)\approx 0.722-0.000\approx 0.722\;\mathrm{bits}\\ \mathrm{MN}(\mathrm{with}\mathrm{negative}\mathrm{bias}): & \;H\left(Z_{\mathrm{cmdc}}^{\mathrm{PM}}\right)-H\left(Z_{\mathrm{cmdc}}^{\mathrm{MN}}\right)\approx 0.722-1.000\approx -0.278\;\mathrm{bits} \end{array}$$

Here, it is useful for us to observe the **potential distortion** (i) between each committee member’s view and the ground truth, and (ii) between each committee member’s view and the proposal.

$$\begin{array}{cc} \mathrm{PM}(\mathrm{the}\mathrm{proposer}): & \;H_{\max}\left(Z_{\mathrm{cmdc}}\right)D\left(Z_{\mathrm{cmdc}}^{*}∥Z_{\mathrm{cmdc}}^{\mathrm{PM}}\right)\approx 7.814\times 0.791\approx 6.182\;\mathrm{bits}\\ \mathrm{MS}(\mathrm{with}\mathrm{soft}\mathrm{knowledge}): & \;H_{\max}\left(Z_{\mathrm{cmdc}}\right)D\left(Z_{\mathrm{cmdc}}^{*}∥Z_{\mathrm{cmdc}}^{\mathrm{MS}}\right)\approx 7.814\times 0.014\approx 0.112\;\mathrm{bits}\\ \mathrm{MP}(\mathrm{with}\mathrm{positive}\mathrm{bias}): & \;H_{\max}\left(Z_{\mathrm{cmdc}}\right)D\left(Z_{\mathrm{cmdc}}^{*}∥Z_{\mathrm{cmdc}}^{\mathrm{MP}}\right)\approx 7.814\times 1.000\approx 7.814\;\mathrm{bits}\\ \mathrm{MN}(\mathrm{with}\mathrm{negative}\mathrm{bias}): & \;H_{\max}\left(Z_{\mathrm{cmdc}}\right)D\left(Z_{\mathrm{cmdc}}^{*}∥Z_{\mathrm{cmdc}}^{\mathrm{MN}}\right)\approx 7.814\times 0.322\approx 2.515\;\mathrm{bits}\\ \mathrm{PM}(\mathrm{the}\mathrm{proposer}): & \;H_{\max}\left(Z_{\mathrm{cmdc}}\right)D\left(Z_{\mathrm{cmdc}}^{\mathrm{PM}}∥Z_{\mathrm{cmdc}}^{\mathrm{PM}}\right)\approx 7.814\times 0=0\;\mathrm{bits}\\ \mathrm{MS}(\mathrm{with}\mathrm{soft}\mathrm{knowledge}): & \;H_{\max}\left(Z_{\mathrm{cmdc}}\right)D\left(Z_{\mathrm{cmdc}}^{\mathrm{PM}}∥Z_{\mathrm{cmdc}}^{\mathrm{MS}}\right)\approx 7.814\times 0.677\approx 5.290\;\mathrm{bits}\\ \mathrm{MP}(\mathrm{with}\mathrm{positive}\mathrm{bias}): & \;H_{\max}\left(Z_{\mathrm{cmdc}}\right)D\left(Z_{\mathrm{cmdc}}^{\mathrm{PM}}∥Z_{\mathrm{cmdc}}^{\mathrm{MP}}\right)\approx 7.814\times 0.0566\approx 0.442\;\mathrm{bits}\\ \mathrm{MN}(\mathrm{with}\mathrm{negative}\mathrm{bias}): & \;H_{\max}\left(Z_{\mathrm{cmdc}}\right)D\left(Z_{\mathrm{cmdc}}^{\mathrm{PM}}∥Z_{\mathrm{cmdc}}^{\mathrm{MN}}\right)\approx 7.814\times 0.452\approx 3.533\;\mathrm{bits} \end{array}$$

It is in fact more important for us to observe the overall view of the committee, which is denoted as $Z_{\mathrm{cmdc}}^{1}$ and is a probabilistic combination of $Z_{\mathrm{cmdc}}^{\mathrm{PM}}$, $Z_{\mathrm{cmdc}}^{\mathrm{MS}}$, $Z_{\mathrm{cmdc}}^{\mathrm{MP}}$, and $Z_{\mathrm{cmdc}}^{\mathrm{MN}}$:

$$\begin{array}{cc} P(Z_{\mathrm{cmdc}1}^{1}) & =\left\{\begin{array}{cc} 0.450 & \mathrm{letter}\;(\mathrm{strong}\;\mathrm{decline},\;\mathrm{one}\;\mathrm{short-term}\;\mathrm{catalyst},\;\mathrm{high}\;\mathrm{conviction})\\ 0.050 & \mathrm{letter}\;(\mathrm{strong}\;\mathrm{decline},\;\mathrm{only}\;\mathrm{one}\;\mathrm{medium-term}\;\mathrm{catalyst},\;\mathrm{high}\;\mathrm{conviction})\\ 0.125 & \mathrm{letter}\;(\mathrm{rise},\;\mathrm{multiple}\;\mathrm{catalysts},\;\mathrm{high}\;\mathrm{conviction})\\ 0.350 & \mathrm{letter}\;(\mathrm{rise},\;\mathrm{multiple}\;\mathrm{catalysts},\;\mathrm{medium}\;\mathrm{conviction})\\ 0.025 & \mathrm{letter}\;(\mathrm{rise},\;\mathrm{multiple}\;\mathrm{catalysts},\;\mathrm{low}\;\mathrm{conviction})\\ 0 & \mathrm{other}\;70\;\mathrm{letters} \end{array}\right.\\ P(Z_{\mathrm{cmdc}2}^{1}) & =\left\{\begin{array}{cc} 0.5 & \mathrm{letter}\;\mathrm{accept}\\ 0.5 & \mathrm{letter}\;\mathrm{reject}\\ 0 & \mathrm{letter}\;\mathrm{request}\;\mathrm{more}\;\mathrm{information} \end{array}\right. \end{array}$$

The committee view at this stage, i.e., $Z_{\mathrm{cmdc}}^{1}$, is less certain (i.e., less *alphabet compression*) but may be closer or further away from the ground truth $Z_{\mathrm{cmdc}}^{*}$ than individual views, i.e., $Z_{\mathrm{cmdc}}^{\mathrm{PM}}$, $Z_{\mathrm{cmdc}}^{\mathrm{MS}}$, $Z_{\mathrm{cmdc}}^{\mathrm{MP}}$, and $Z_{\mathrm{cmdc}}^{\mathrm{MN}}$:

$$\begin{array}{cc} \mathrm{alphabet}\mathrm{compression}(\mathrm{committee}): & \;H_{\max}\left(Z_{\mathrm{cmdc}}\right)-H\left(Z_{\mathrm{cmdc}}^{1}\right)\approx 7.814-1.773\approx 6.041\;\mathrm{bits}\\ \mathrm{potential}\mathrm{distortion}(\mathrm{committee}): & \;H_{\max}\left(Z_{\mathrm{cmdc}}\right)D\left(Z_{\mathrm{cmdc}}^{*}∥Z_{\mathrm{cmdc}}^{1}\right)\approx 7.814\times 0.405\approx 3.161\;\mathrm{bits} \end{array}$$

Like most committee-based decision processes, the members of the committee spend time to deliberate on their respective views. This can be modeled as the progression of the committee alphabet from $Z_{\mathrm{cmdc}}^{0}$ to $Z_{\mathrm{cmdc}}^{1}$, then $Z_{\mathrm{cmdc}}^{1.1}$, $Z_{\mathrm{cmdc}}^{1.2}$, $Z_{\mathrm{cmdc}}^{1.3}$, …. Ultimately, the committee reaches a decision. In this case study, it is reasonable to assume that the members of the committee gradually are largely convinced by the arguments of **MS** (the member with soft knowledge), leading to the final committee decision alphabet, $Z_{\mathrm{cmdc}}^{2}$, as follows:

$$\begin{array}{cc} P(Z_{\mathrm{cmdc}1}^{2}) & =\left\{\begin{array}{cc} 0.8 & \mathrm{letter}\;(\mathrm{rise},\;\mathrm{multiple}\;\mathrm{catalysts},\;\mathrm{medium}\;\mathrm{conviction})\\ 0.2 & \mathrm{letter}\;(\mathrm{rise},\;\mathrm{multiple}\;\mathrm{catalysts},\;\mathrm{low}\;\mathrm{conviction})\\ 0 & \mathrm{other}\;73\;\mathrm{letters} \end{array}\right.\\ P(Z_{\mathrm{cmdc}2}^{2}) & =\left\{\begin{array}{cc} 1 & \mathrm{letter}\;\mathrm{reject}\\ 0 & \mathrm{letter}\;\mathrm{request}\;\mathrm{more}\;\mathrm{information} \end{array}\right. \end{array}$$

The final decision alphabet of the committee, $Z_{\mathrm{cmdc}}^{2}$, is more certain (that is, more *alphabet compression*) and is closer to the ground truth $Z_{\mathrm{cmdc}}^{*}$ in comparison with $Z_{\mathrm{cmdc}}^{0}$ and $Z_{\mathrm{cmdc}}^{1}$:

$$\begin{array}{cc} \mathrm{alphabet}\mathrm{compression}(\mathrm{committee}): & \;H_{\max}\left(Z_{\mathrm{cmdc}}\right)-H\left(Z_{\mathrm{cmdc}}^{2}\right)\approx 7.814-0.722\approx 7.092\;\mathrm{bits}\\ \mathrm{potential}\mathrm{distortion}(\mathrm{committee}): & \;H_{\max}\left(Z_{\mathrm{cmdc}}\right)D\left(Z_{\mathrm{cmdc}}^{*}∥Z_{\mathrm{cmdc}}^{2}\right)\approx 7.814\times 0.057\approx 0.442\;\mathrm{bits} \end{array}$$

Therefore, the informative **benefit** for the individual views, i.e., $Z_{\mathrm{cmdc}}^{\mathrm{PM}}$, $Z_{\mathrm{cmdc}}^{\mathrm{MS}}$, $Z_{\mathrm{cmdc}}^{\mathrm{MP}}$, and $Z_{\mathrm{cmdc}}^{\mathrm{MN}}$, and committee views, i.e., $Z_{\mathrm{cmdc}}^{0}$, $Z_{\mathrm{cmdc}}^{1}$, and $Z_{\mathrm{cmdc}}^{2}$ are as follows:

$$\begin{array}{cc} \mathrm{PM}\;(\mathrm{the}\mathrm{proposer}): & \;7.092-6.182\approx 0.910\;\mathrm{bits}\\ \mathrm{MS}\;(\mathrm{with}\mathrm{soft}\mathrm{knowledge}): & \;7.345-0.112\approx 7.233\;\mathrm{bits}\\ \mathrm{MP}\;(\mathrm{with}\mathrm{positive}\mathrm{bias}): & \;7.814-7.814=0\;\mathrm{bits}\\ \mathrm{MN}\;(\mathrm{with}\mathrm{negative}\mathrm{bias}): & \;6.814-2.515\approx 4.299\;\mathrm{bits}\\ \mathrm{CMDC}\;0\;(\mathrm{at}\mathrm{beginning}): & \;7.092-6.182\approx 0.910\;\mathrm{bits}\\ \mathrm{CMDC}\;1\;(\mathrm{before}\mathrm{discussions}): & \;6.041-3.161\approx 2.880\;\mathrm{bits}\\ \mathrm{CMDC}\;2\;(\mathrm{final}\mathrm{decision}): & \;7.092-0.442\approx 6.650\;\mathrm{bits} \end{array}$$

We estimate the costs of the committee in terms of the amount of time that they would be expected to spend during the committee meeting to make their decision. Assuming the investment committee meeting took 1.5 h, comprised of 15 min for the proposal presentation, 30 min for each member to share their initial views, and 45 min for discussions and final decisions, we estimate the cost–benefit ratios of the three committee stages as:

**CMDC 0****CMDC 1****CMDC 2**$\frac{\mathrm{Benefit}}{\mathrm{Cost}}$$\frac{0.910}{0.250}=3.640\;\frac{\mathrm{bits}}{\mathrm{hour}}$$\frac{2.880}{0.500}=5.760\;\frac{\mathrm{bits}}{\mathrm{hour}}$$\frac{6.650}{0.750}=8.867\;\frac{\mathrm{bits}}{\mathrm{hour}}$as well as the gradual reduction in potential distortion of the committee alphabet as follows:

$$D\left(Z_{\mathrm{cmdc}}^{*}\;∥\;Z_{\mathrm{cmdc}}^{0}\right)>D\left(Z_{\mathrm{cmdc}}^{*}\;∥\;Z_{\mathrm{cmdc}}^{1}\right)>D\left(Z_{\mathrm{cmdc}}^{*}\;∥\;Z_{\mathrm{cmdc}}^{2}\right)$$

This highlights the merits of such a committee, although it also had biased committee members and an initially inappropriate proposal. We conclude that the investment committee process is cost-beneficial based on the information-theoretic analysis. As the investment committee meeting progresses from $Z_{\mathrm{cmdc}}^{0}$ to $Z_{\mathrm{cmdc}}^{2}$, the informative benefit increases and the potential distortion decreases at every stage. As long as the discussions remain fact-based among the committee members, they can result in a committee PMF convergence of $Z_{\mathrm{cmdc}}$ towards the ground truth PMF of $Z_{\mathrm{cmdc}}^{*}$ due to the soft knowledge and a mitigation of positive and negative biases.

## 5. Discussions

In this section, we first summarize and discuss the findings of the three case studies. We then consider the difference between economic cost–benefit analysis and information-theoretic cost–benefit analysis. This is followed by discussions on several related research topics, which present both challenges and opportunities in further development of the work reported in this paper.

### 5.1. The Findings of Three Case Studies

As this work aims to translate an information-theoretic concept and measure for cost–benefit analysis to a methodology that is relevant to business and finance, the findings of the three case studies are indicative within the scope of conceptual and methodological research. We present the general findings first, followed by the findings of each case study.

**General:**

1. The three case studies confirmed that the information-theoretic concept can be used to analyze the effectiveness of information transformations in various component processes in decision workflows in business and finance. The effectiveness, referred to as “benefit”, has two measures, alphabet compression and potential distortion. On the one hand, such transformations should ideally achieve as much alphabet compression as possible. On the other hand, they should ideally have as little potential distortion as possible. Hence, optimizing a process involves finding the optimal trade-off between these two measures.
2. As shown in Figure 1, the processes in the top-level workflow are too complex (i.e., too many PMFs to consider) or too vague (the PMFs are not well-defined) for us to conduct information-theoretic analysis. Therefore, it is necessary to decompose each process into several low-level processes. The decomposition procedure can be recursively invoked until each process is defined with a small number of PMFs. This is a typical engineering approach for workflow modeling and decomposition. The three case studies confirmed that this approach can also be applied to workflows in business and finance.
3. The three case studies feature one machine-centric process (i.e., Case Study 1) and two human-centric processes (i.e., Case Studies 2 and 3). They confirmed that the methodology can be applied to both machine- and human-centric processes.
4. The computation reported in the three case studies was carried out using tools such as spreadsheets. While this approach is effective in conceptual and methodological studies, it does not scale up to real-world workflows, where there are numerous processes and many PMFs. It is thus highly desirable to develop software tools for supporting such computation.

**Case Study 1:**

5. This case study indicates that machine-centric processes usually produce very high alphabet compression at a low cost (excluding the development cost of the process).
6. Some machine-centric processes have the risk of incurring high potential distortion.
7. Data visualization, commonly used in business and finance, provides an effective means for alleviating the potential distortion. Data visualization usually reduces entropy less than statistics and algorithms, and the cost of using data visualization is relatively low.

**Case Study 2:**

8. This case study demonstrates that information-theoretic cost–benefit analysis can be used to examine scenarios of missing information in data and additional information provided through human knowledge (i.e., the instance of a diligent portfolio manager).
9. Information-theoretic analysis can quantitatively demonstrate that missing information may lead to higher potential distortion, thereby reducing the overall cost–benefit measure.
10. Information-theoretic analysis can quantitatively show that soft knowledge can reduce potential distortion, increasing the informative benefit.

**Case Study 3:**

11. This case study demonstrates that information-theoretic cost–benefit analysis can be used to examine scenarios that feature soft knowledge, as well as positive and negative biases.
12. Information-theoretic analysis can quantitatively show that both positive and negative bias increase potential distortion, thereby reducing the cost–benefit of the process.
13. Information-theoretic analysis can quantitatively demonstrate that committee meetings can be effective if they include members with useful soft knowledge.
14. Information-theoretic analysis can quantitatively show that committee meetings can be effective if there is a reasonable balance between members with positive and those with negative bias.

### 5.2. Comparison with Traditional Economic CBA Methods

Traditional economic CBA methods (e.g., [12,15,45]) focus on monetary outcomes over time and seek beneficial risk-adjusted returns. Many of these methods have been extensively studied and are now widely used in business and finance. The work reported in this paper is not intended to replace any of these methods, but to address a gap that these methods cannot cover.

Today, many decision workflows involve both machine- and human-centric processes. At a higher level, one may use “monetary benefit” and “monetary cost” to characterize these processes and economic CBA methods are still applicable. At a lower level, each process receives raw data, processed data from preceding processes, and/or knowledge from humans, while generating data to be processed by the succeeding process. Using “monetary benefit” to characterize the benefit of such information transformations is rather coarse, to say the least.

Information-theoretic cost–benefit analysis offers a potential to analyze machine- and human-centric processes in hybrid decision workflows at such a lower level. Some may argue that such low-level analysis is unnecessary. This reminds us of the time when the concept of entropy was first proposed by Ludwig Boltzmann in 1877. Many scientists then considered that the modeling of thermodynamic phenomena using a concept based on microscopic behaviors was unnecessary, and the macroscopic measures (e.g., temperature, volume, density, etc.) were adequate. Today, the concept of entropy underpins not only thermodynamics but also information theory, data communication, and many computational subjects.

Meanwhile, we must recognize that economic CBA methods have been around for decades while Information-theoretic cost–benefit analysis was first proposed in 2016 [33]. There is no doubt a long road ahead to reach its maturity theoretically and to be developed into a technology that can be deployed in practical applications for optimizing hybrid decision workflows in business and finance.

### 5.3. Estimation of Probability Distribution, Uncertainty Analysis, and Sensitivity Analysis

This work focuses on the translation from the information-theoretic cost–benefit analysis to a methodology for analyzing hybrid decision workflows in business and finance. For this work, we assumed that all probability mass functions (PMFs) in the analysis have already been obtained. In practice, obtaining accurate PMFs is a significant challenge, and there is a large volume of previous work on PMF and entropy estimation (e.g., [46,47,48]), uncertainty analysis (e.g., [49,50,51,52]), sensitivity analysis (e.g., [53,54,55]), and interval computation (e.g., [56,57,58]). The conceptual work reported in this paper should be considered as an early step towards deployable software tools and real-world applications. Like many previous developments that transitioned from theoretical concepts to practical applications in business and finance, there will be numerous steps of technical advancement by researchers in the field. Such technical advancement will be necessary to address several challenges, which include, but are not limited to, the following:

- *PMFs Estimation*—The credibility of information-theoretical analysis depends critically on the accuracy of the PMFs available at every stage of the analytical process. PMFs can be estimated from various types of data, including measured real-world data, survey data, and experimental data. To analyze a hybrid decision workflow, one has to consider a number of challenges, including frequent temporal changes to PMFs, global PMFs vs. local PMFs, sparsity samples of human decision patterns, and so on.
- *Uncertainty Analysis*—The uncertainty of a univariate variable is often modeled as a distribution of the possible values of the variable. One can easily anticipate the complexity of modeling the uncertainty of a PMF, since there would be distributions of distributions. The complexity increases rapidly as the number of letters in a PMF and/or the number of PMFs whose interactions need to be analyzed grows.
- *Sensitivity Analysis*—With information-theoretic analysis, changes to some PMFs often have more impact than changes to other PMFs. Sensitivity analysis allows one to identify important variables that contribute critically to the outcome (e.g., used in COVID-19 modeling [59]). Similar to uncertainty analysis, the sensitivity analysis of *n* PMFs is, in general, much more complex than that of *n* variables.
- *Interval Computation*—Instead of specifying the distributions of each probability element $p_{i}$ in a PMF, one may use a simplified way to select the error bounds of each $p_{i}$. Interval computation is a family of methods for computing with error bounds. The application of Interval computation to PMFs usually is more complex than that with multiple variables, as each PMF may have different error bounds for each $p_{i}$ in a PMF and the computation needs to maintain the validity of every PMF (i.e., $p_{i}\in \left[0,1\right]$ and $\sum p_{i}=1$).
- *Software Tools*—With the aforementioned challenges, one can anticipate that future research will be able to formulate mathematical solutions. To make these mathematical solutions practically useful, the key will be to develop software tools that enable the automation of such computations.

These challenges cannot be addressed by any single piece of research, but require much extensive research effort in the future.

## 6. Conclusions and Future Directions

In this paper, we presented a methodology for analyzing the cost–benefit of processes in data-informed decision workflows. The methodology is based on the theoretical development of information-theoretic cost–benefit analysis, as well as a practical approach to workflow decomposition in engineering. Using three case studies, we demonstrate the feasibility of conducting such an analysis quantitatively. This confirms the main research question about whether we can translate the information-theoretic concept and measure for cost–benefit analysis to a methodology for analyzing hybrid decision workflows in business and finance. It also confirms that the engineering approach for workflow modeling and decomposition is a valid and useful instrument for enabling the translation.

As three case studies feature machine- and human-centric processes (including a statistical and computational algorithm, incomplete information and human soft knowledge, and human biases in a committee meeting), they also demonstrate the general applicability of the proposed methodology.

We recognize that the method is currently not automated or supported by any computer-assisted tool. This is a typical limitation of any quantitative method that has not yet been supported by a computer-assisted tool. We strongly believe that future research will lead to the development of computer-assisted tools by building on past, current, and future theoretical, conceptual, and methodological research on information-theoretic cost–benefit analysis, including this work. Meanwhile, we will continue our methodological research by applying information-theoretic cost–benefit analysis to processes in machine learning workflows.

## Data Availability

The data and calculations for the case studies are presented in the paper.

## Abbreviations

The following abbreviations are used in this manuscript:

AC	Alphabet Compression
Bel	Believed
CBA	Cost–Benefit Analysis
Cmdc	Committee Decision
CS	Case Study
Ct	Cost
JS	Jenson-Shannon
KL	Kullback–Leibler
MA	Simple Moving Average
MDPs	Markovian Decision Processes
Miss	Missing Information
MN	Committee Member with Negative Bias
MP	Committee Member with Positive Bias
MS	Committee Member with Soft Knowledge
PD	Potential Distortion
PM	Portfolio Manager
PMF	Probability Mass Function
Ref	Reference
Rcmd	Recommendations
Sv	Share Value
Vis	Visualization
XAI	Explainable Artificial Intelligence
